# Diabetogenic Effects of Ochratoxin A in Female Rats

**DOI:** 10.3390/toxins9040144

**Published:** 2017-04-19

**Authors:** Firdevs Mor, Omur Sengul, Senay Topsakal, Mehmet Akif Kilic, Ozlem Ozmen

**Affiliations:** 1 Department of Pharmacology and Toxicology, Faculty of Veterinary Medicine, Mehmet Akif Ersoy University, Istiklal Yerleskesi, Burdur 15030, Turkey; omursengul@mehmetakif.edu.tr; 2 Department of Endocrinology and Metabolism, Faculty of Medicine, Pamukale University, Denizli 20070, Turkey; stopsakal@hotmail.com; 3 Division of Biology, Faculty of Science, Akdeniz University, Antalya 07058, Turkey; mkilic@akdeniz.edu.tr; 4 Department of Pathology, Istiklal Yerleskesi, Faculty of Veterinary Medicine, Mehmet Akif Ersoy University, Burdur 15030, Turkey; ozlemozmen@mehmetakif.edu.tr

**Keywords:** Ochratoxin A, insulin, glucagon, glucose, rat plasma, pathology, immunohistochemistry

## Abstract

In this study, the diabetogenic effects of long term Ochratoxin A (OTA) administration in rats were investigated, and its role in the etiology of diabetes mellitus (DM) was examined utilizing 42 female Wistar rats for these purposes. The rats were divided into three different study and control groups according to the duration of the OTA administration. The rats received 45 μg OTA daily in their feed for 6, 9 and 24 weeks, respectively. Three control groups were also used for the same time periods. Blood and pancreatic tissue samples were collected during the necropsy at the end of the 6, 9 and 24 weeks. The plasma values of insulin, glucagon and glucose were determined for the study and control groups. Pancreatic lesions were evaluated via histopathological examination and insulin and glucagon expression in these lesions was subsequently determined using immunohistochemical methods. Statistically significant decreases in insulin levels were observed, in contrast to increases in blood glucagon and glucose levels. Histopathological examinations revealed slight to moderate degeneration in Langerhans islet cells in all OTA-treated groups. Immunohistochemistry of pancreatic tissue revealed decreased insulin and increased glucagon expression. This study demonstrated that OTA may cause pancreatic damage in the Langerhans islet and predispose rats to DM.

## 1. Introduction

Ochratoxin A (OTA) is a mycotoxin that naturally occurs as a fungal metabolite and is the most toxic product of *Aspergillus ochraceus* and *Penicillium verrucosum* [1]. Its widespread occurrence in human and animal food in conjunction with some preliminary cytotoxic and carcinogenic data suggest a possible role for dietary OTA in the development of various organ damage and the occurrence of different tumors [2,3,4,5]. In addition to carcinogenic, mutagenic and cytotoxic effects, OTA is also known to lead to a decrease in food intake and body weight gain [6]. OTA has been shown to be nephrotoxic, immunotoxic and teratogenic to a variety of animal species [7]. It is a ubiquitous mycotoxin produced by fungi in improperly stored food products. Various commodities including corn, peanuts, wheat, maize, rye, barley, coffee beans, flour, rice, spices, bread and animal feed may contain OTA. The highest amounts of OTA in food of plant origin were found mainly in Eastern Europe [8,9,10,11,12].

Diabetes mellitus (DM) is an autoinflammatory syndrome that is a collection of many disorders such as hyperglycemia, dyslipidemia, insulin resistance, impaired beta-cell functioning, and insulin secretion [13,14,15,16]. DM is associated with disturbance of carbohydrate, fat, and protein metabolism resulting from defects in insulin secretion, insulin action, or both [17]. It is considered to be among the major life-threatening diseases worldwide, particularly in developing countries [13,14,15,16]. DM is one of the most crippling diseases that humankind has ever had to deal with, and its prevalence has risen dramatically over the past two decades [18]. Currently, there are over 150 million diabetics worldwide, and this is likely to increase to 300 million or more by the year 2025 due to increased sedentary lifestyle, consumption of energy-rich diet, and obesity [19].

There are few studies investigating the diabetogenic effects of OTA, the extent of which is far beyond clear. In particular, increased DM incidence in animals necessitates the clarification of any possible relation between OTA consumption and occurrence of DM. The design of this study was based on the increased incidence of OTA in contaminated foods and DM in underdeveloped countries, which indicates possible relations between OTA and diabetes. The aim of this study was to examine OTA toxicity in the pancreas and the possible diabetogenic effects of this toxication in a rat model.

## 2. Results

**Figure 1:**
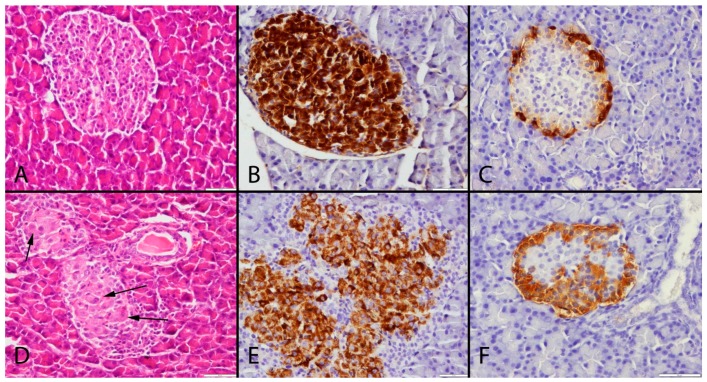
Histopathological and immunohistochemical appearance of the pancreas after a 24-week period. (**A**) normal histology, Hematoxylin and Eosin; (**B**) severe insulin; and (**C**) glucagon immunoexpression in the control group, streptavidin biotin peroxidase method; (**D**) degenerative and karyomegalic cells in the Langerhans islet (indicated by the arrows), Hematoxylin and Eosin; (**E**) decreased insulin; and (**F**) increased glucagon expression, streptavidin biotin peroxidase method, bar = 50 μm.

All pancreases had normal gross appearance during necropsy in all groups. Histopathological examinations of rats revealed vacuolization, megalocytosis and karyomegaly in some islets of Langerhans in the OTA-treated groups (OTA6, OTA9 and OTA24) while no pathological lesions were observed in the control groups (Ctrl6, Ctrl9 and Ctrl24) (Figure 1A,D).

**Figure 2:**
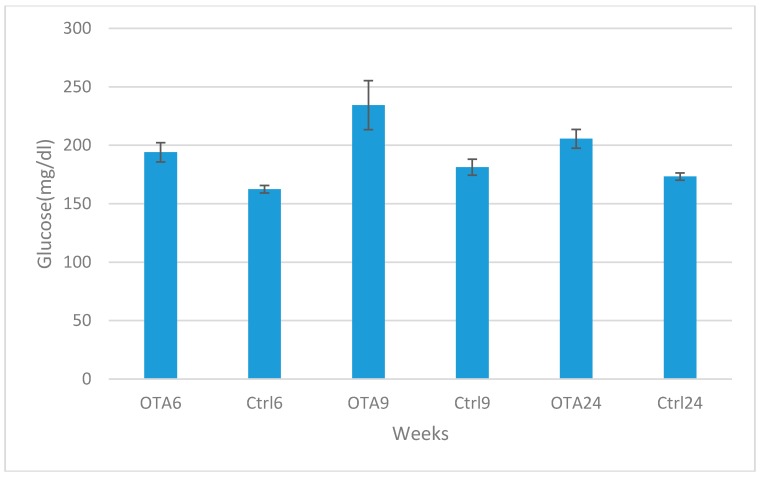
Statistical analysis of plasma glucose levels in the study and control groups. Statistically significant increases were observed in the study group results when compared to the same periods in the control groups (*p* = 0.009 in OTA6 and Ctrl6; *p* = 0.048 in OTA9 and Ctrl9; *p* = 0.007 in OTA24 and Ctrl24).

**Figure 3:**
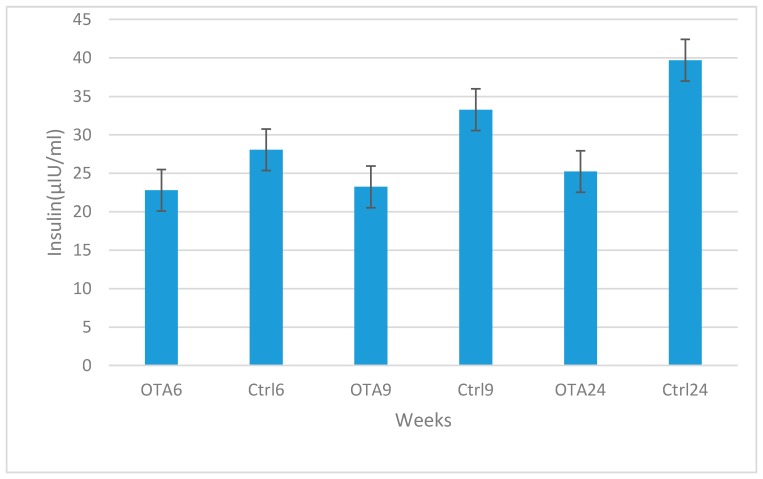
Statistical analysis of insulin levels in the study and control groups. Statistically significant decreases were observed in the study group results when compared to the same periods in the control groups (*p* = 0.048 in OTA6 and Ctrl6; *p =* 0.010 in OTA9 and Ctrl9; *p =* 0.035 in OTA24 and Ctrl24).

**Figure 4:**
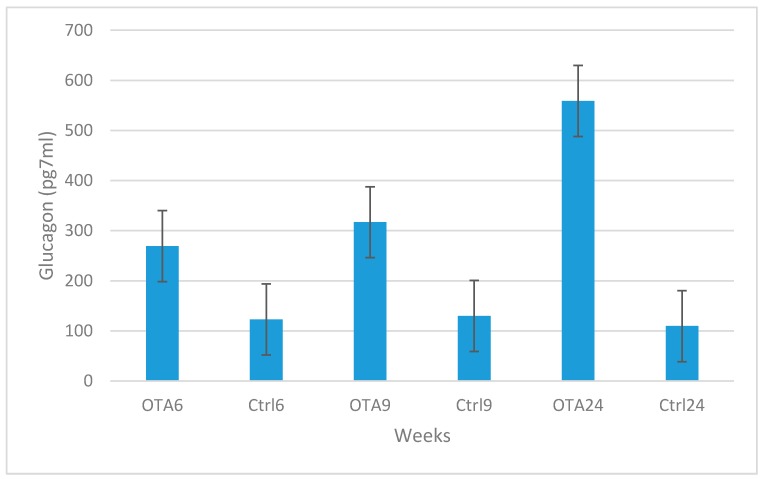
Statistical analysis of plasma glucagon levels in the study and control groups. Statistically significant increases were observed in the study group results when compared to the same periods in the control groups (*p =* 0.007 in OTA6 and Ctrl6; *p =* 0.035 in OTA9 and Ctrl9; *p =* 0.004 in OTA24 and Ctrl24).

Marked increases were observed in blood glucose and glucagon levels in contrast to decreases in insulin levels in the study groups compared to the control groups in 6, 9 and 24-week study periods. Statistical analysis results of plasma glucose, insulin and glucagon levels are shown in Figure 2, Figure 3 and Figure 4. Statistically significant differences were observed in all study periods between study and control groups. This study showed that OTA causes damage in endocrine pancreatic function, even during a six-week exposure period. 

The immunohistochemical examinations showed that the insulin-secreting cells were localized in the central area of the islets of Langerhans. Marked insulin secretion was observed in the control groups, and the OTA treatment caused a slight to moderate decrease in the number of insulin-secreting cells together with the degree of insulin expression in some Langerhans islets. Furthermore, the decrease in insulin expression is augmented with the increased duration of OTA exposure (Figure 1B,E).

**Figure 5:**
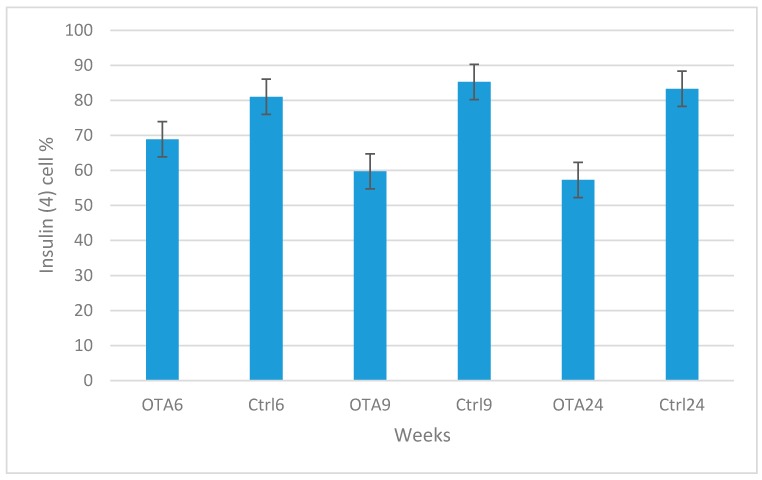
Statistical analysis of insulin positive cell percentages in the groups. Statistically significant decreases were seen in the study group results compared to the same period in the control groups (*p =* 0.004 in OTA6 and Ctrl6; *p* < 0.001 in OTA9 and Ctrl9; *p* < 0.001 in OTA24 and Ctrl24).

**Figure 6:**
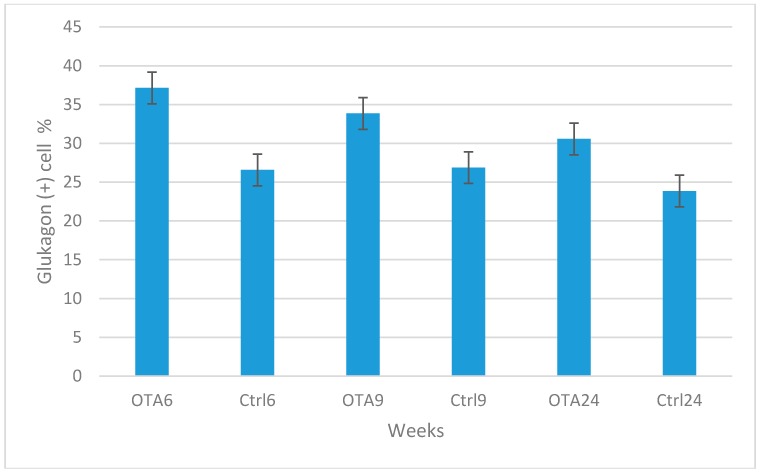
Statistical analysis of glucagon positive cell percentages in the groups. Statistically significant increases were seen in the study group results compared to the same period in the control groups (*p* < 0.001 in OTA6 and Ctrl6; *p* = 0.025 in OTA9 and Ctrl9; *p =* 0.048 in OTA24 and Ctrl24).

Similar levels of glucagon expression were observed in the control groups during the study, whereas a slight increase occurred in the OTA-treated groups (Figure 1C,F). However, glucagon positive cells were decreased with the increased duration of OTA exposure. The results of the statistical analysis of the numbers of insulin and glucagon expressing cells are shown in Figure 5 and Figure 6.

## 3. Discussion

OTA toxication and DM are increasing problems in both human and animal health. There appears to be a link between OTA toxication and DM, as both are related to food consumption. A few studies have reported OTA as the possible cause of endocrine pancreas problems, but the diabetogenic effects of OTA are unclear. The purpose of this study was to examine OTA toxicity in the pancreas and the possible diabetogenic effects of this toxication in a rat model.

DM is a common illness with high morbidity and an early mortality rate, causing vascular, renal, retinal, or neuropathic disorders in the long term as well as acute metabolic complications [20]. Diet along with lifestyle modification is believed to play an important role in the management of this disease [21,22]. Polydipsia and polyphagia are the most common symptoms of diabetes [17]. OTA is a ubiquitous mycotoxin produced by the fungi of improperly stored food products [23]. Because both OTA-related toxicity and DM are food intake-related diseases, the idea of any possible relation between these conditions was the main objective of this study.

There is little knowledge about the relationships between mycotoxins and DM [24,25,26]. These authors reported increases in glucose levels after mycotoxin exposure in rats and chickens. Similar increases in blood glucose levels were observed in OTA-treated groups in this study. However, there is no report detailing pathological and histopathological findings in OTA toxication. This is the first study on the effects of OTA on the pancreas in a rat model. 

Suseela et al., 1986, reported increased glucose and decreased insulin levels following mycotoxin administration in rats [26]. Subramanian et al., 1985, also reported similar results in calves after OTA exposure [25]. In a recent study, after 30 days of OTA exposure, liver and kidney damages were also reported in addition to increases in blood glucose levels [27]. The results of our study are in agreement with previous studies revealing OTA as the cause of increases in blood glucose and glucagon levels, as well as decreases in blood insulin levels in groups that are administered OTA-contaminated food for all of the given time periods. 

Changes in blood glucose levels as a result of OTA exposure have been reported in pigs [28,29,30,31], rabbits [32] and rats [27,33]. Studies with pigs on blood glucose levels following OTA toxication are contradictory [28,29,30,31]. Decreased blood glucose levels were observed in rabbits fed with 1 and 2 ppm OTA-contaminated food after three weeks and, at week 8, blood glucose levels were half those of the control group [32]. These seemingly contradictory and inconclusive results could be related to the wide range of OTA doses used in these studies and the exposure periods as well as the possible species-specific effect of OTA exposure. On the other hand, there have been two studies reporting that OTA can cause increases in blood glucose levels in rats. In the first study, OTA (100 g/rat/day) was given orally by gavage for eight weeks; it was found that blood glucose levels increased while insulin levels decreased [33]. Similarly, in the second study, 30 days of OTA treatment resulted in blood glucose level increases [27]. As both studies indicated that OTA is a possible diabetogenic toxin, its direct effects on the pancreas and pancreatic cells were revealed for the first time in addition to its effects on blood glucose, insulin and glucagon levels. In fact, a relationship between OTA and blood glucagon levels and a marked glucagon decrease after six weeks of OTA exposure were also first reported in this study. In addition to all these new findings, the diabetogenic effects of OTA exposure were first observed in a time dependent manner, from six weeks to 24 weeks. Furthermore, this is the first study showing that OTA does not only alter blood glucose and insulin levels in rats, but also leads to increases in blood glucagon levels and causes pancreatic lesions.

A decrease in insulin secretion or diminished insulin activity causes a rise in glucagon concentrations and pancreatic expression of the hormone [32]. Although there are reports on insulin and glucose levels in mycotoxin-administered rats, there is no knowledge regarding glucagon levels in OTA-related rat studies in the literature. This is the first study to examine glucagon levels in a rat model of OTA toxication together with blood results obtained by ELISA and pancreas results by immunohistochemistry. In agreement with a study by Ozmen et al. [34], the results of this study demonstrate that OTA causes an increase in blood glucagon levels and expression in the pancreas. The cause of this result may be related to decreased insulin secretion and a relative increase in glucagon secreted cells. Very sensitive insulin and glucagon ELISA kits were used, and assay results supported the immunohistochemical findings. 

OTA can cause degenerative, necrotic and cancerogenic effects in cells. Karyomegaly is the most common and characteristic pathological finding in liver and kidney cells after OTA exposure [35]. However, there is no knowledge of pancreatic cell reactions after OTA treatment. This study demonstrates that OTA causes degeneration, karyomegaly and megalocytosis in pancreatic cells. Insulin secreted cells were found to be more sensitive to OTA toxication in this study, compared to glucagon secreted cells.

DM is one of the most challenging metabolic disorders, as it cannot be cured but must only be managed. Numerous people and animals suffer from DM throughout the world [36,37]. Numerous studies have been performed on the etiology, pathogenesis and treatment of this significant and common disease. OTA is the main cause of the various organ and tissue damage in exposed humans and animals [2,3,4,5,27]. The results of this study suggest that OTA may also be involved in diabetogenic agents. In this study, none of our three control groups exhibited any abnormality in either plasma levels or pancreatic tissues, therefore all of the abnormal plasma and tissue results can be attributed to OTA toxicity.

The main potential flaw of the study was the small number of rats used. However, because of the long duration and the fact that there were six groups, the number of rats was reduced. The other possible limitation may be that only immunohistochemistry was used for molecular analysis. Further and expanded research may be planned for better evaluation. Because this is the first extensive study of its kind, it will be a basis for further such studies. 

The results of this study have demonstrated that OTA can have toxic effects on the endocrine pancreas in a rat model. Although toxicity can be observed even in a six-week period of exposure, longer term OTA exposure may be more effective. Our results demonstrated that OTA damage in the exocrine pancreas may be an etiologic factor for DM in humans and animals. In addition, our study suggests a possible relationship between the OTA exposure period and the severity of pancreatic damage. This is the first detailed research on diabetogenic effects. Further and expanded studies are needed for the clarification of the relationship between OTA and DM.

## 4. Materials and Methods

### 4.1. Animals, Housing Conditions and Experimental Procedures

In this study, pancreas and plasma samples were collected from our previous study as approved by Akdeniz University Local Animal Research Committee (No: 2011.12.02). The study was performed in accordance with the National Institutes of Health Guidelines for the Care and Handling of Animals. Rats were kept in the experimental animal laboratory of Akdeniz University. This laboratory has an international accreditation certificate attesting suitable conditions for such work.

Female Wistar rats (16 weeks old), from the Akdeniz University Animal Experiment Unit, were housed on sawdust bedding in groups of four to five in polycarbonate cages with stainless steel covers. Room temperature was maintained at 22 ± 2 °C with a relative humidity of 55 ± 10% and a day/night cycle of 12 h. Rats were given a diet (20 g/rat/day) in stainless steel containers, and water was available ad libitum. The physical condition of each rat was assessed daily for any obvious clinical signs.

Forty-two rats, weighing 125–150 g at the start of the experiment, were randomly allocated into six groups (*n* = 7). The experimental groups (OTA6, 9, 24) were given an OTA-contaminated diet for 6, 9 and 24 weeks, respectively. The control groups (Ctrl6, 9 and 24) were given the standardized powdered rat diet for the same period of time. The feeding methods and OTA doses were essentially the same as those described by Mor et al. (2014). Rats were given 3 mg OTA kg^−1^ food and the initial OTA dose was approximately 45 g OTA/rat/day.

At the end of the study periods, blood (1–1.5 mL) was collected from a tail vein under ether anesthesia, centrifuged immediately and the plasma stored at −40°C. After blood collection, rats were euthanized to collect tissue samples.

### 4.2. Plasma Insulin, Glucagon and Glucose Determination

Plasma glucose levels were analyzed using an Autoanalyzer (Gesan chem 200 Gesan Production srl, Campobello, Italy). Plasma insulin and glucagon levels were analyzed using an ultrasensitive rat insulin ELISA kit (Cat. No: Rab 3050; Sigma Aldich Chemie GmBH, St. Louis, MO, USA) and glucagon ELISA kit (Cat. No: Rab 0202; Sigma Aldich Chemie GmBH, St. Louis, MO, USA) with a multiplate ELISA reader (EPOCH microplate reader; Bio-Tek, Inc., Winooski, VT, USA). Results are expressed as mg/dL for glucose, μIU/mL for insulin and pg/mL for glucagon.

The minimum detectable amount (MDA) for rat insulin was 5 μIU/mL. Recovery was performed by spiking plasma samples with rat insulin (10–50 μIU/mL) and the recovery was 67–129% with an average of 94.68%.

The minimum detectable amount (MDA) of glucagon was 6.37 pg/mL. The recovery of glucagon spiked to levels throughout the range of the assay in rat plasma. The mean recovery was 93% and the range was 86–101%.

The methods used for plasma insulin and glucagon levels were in accordance with the manufacturer’s instructions.

### 4.3. Histological Examination 

During the necropsy, pancreatic tissue samples were collected and fixed in 10% buffered formalin. After routine processing, tissues were embedded in paraffin and sectioned to a thickness of 5 μm. Tissue sections were stained with hematoxylin-eosin and examined microscopically.

Pancreas samples were then immunostained with insulin antiserum (Anti-insulin + Proinsulin antibody, [D6D4] Abcam (ab8304), 1/100 dilution; Abcam, Cambridge, UK) and glucagon antiserum (antiglucagon antibody, Abcam (ab8055), 1/100 dilution; Abcam, Cambridge, UK) according to the manufacturer’s instructions. All of the slides were analyzed for hormone positivity and a semi-quantitative analysis was carried out as detailed later. The overall number of positive cells in 1 high-power field (40×), as well as the number of cells per islet that were positive for each hormone, were noted and compared with normal pancreatic tissue counts. An attempt was made to quantify the percentage positivity of each hormone-producing cell in each of the islets. All islets in at least 5 high-power fields were selected, the total number of nuclei in each islet was counted, and the average number of nuclei/islet was calculated. The average percentage of cells positive for each hormone was then calculated for the islets. Morphometric evaluation was made by using the Database Manual Cell Sens Life Science Imaging Software System (Olympus Corporation, Tokyo, Japan).

### 4.4. Statistical Analysis

One-way ANOVA analysis of variance tests were used to determine whether there were any significant differences between the groups with regard to their blood glucose, glucagon and insulin values. In the determination of differences between groups, the Bonferroni Dunn test was used. Calculations were made using the MiniTab (2011) program pack.
